# First record of *Paegniodes* Eaton, 1881 (Ephemeroptera, Heptageniidae) from Thailand with description of a new species

**DOI:** 10.3897/zookeys.1036.64880

**Published:** 2021-05-10

**Authors:** Boonsatien Boonsoong, Chonlakran Auychinda, Michel Sartori, Nuttakun Khanyom

**Affiliations:** 1 Animal Systematics and Ecology Speciality Research Unit (ASESRU), Department of Zoology, Faculty of Science, Kasetsart University, Bangkok 10900, Thailand Kasetsart University Bangkok Thailand; 2 Department of Biology and Health Science, Mahidol Wittayanusorn School, Salaya, Nakhon Pathom, Thailand Mahidol Wittayanusorn School Nakhon Pathom Thailand; 3 Museum of Zoology, Palais de Rumine, Place Riponne 6, CH-1005 Lausanne, Switze rland Museum of Zoology Lausanne Switzerland; 4 University of Lausanne (UNIL), Department of Ecology and Evolution, CH-1015 Lausanne, Switzerland University of Lausanne Lausanne Switzerland

**Keywords:** COI, diversity, mayfly, Southeast Asia

## Abstract

A new species of Heptageniidae, *Paegniodes sapanensis***sp. nov.**, is described based on larvae, subimagos, eggs, and COI data. The mayfly genus *Paegniodes* Eaton, 1881 is reported for the first time from Thailand. The larva of the new species can be distinguished from other known *Paegniodes* species by i) lamellae of gill I ca 1/4 of fibrilliform portion and ii) mandibles and basal segment of maxillary palp without dense setae on margin. The subimago characters useful to distinguish this new species from previously known species are i) the median stripes on abdominal terga and ii) shape of the female subgenital and subanal plates. The genetic distance between the new species and *P. cupulatus* (Eaton, 1871) was approximately 11%. The morphological characters of the new species are discussed and compared to other known species.

## Introduction

The poorly known mayfly genus *Paegniodes*, established by Eaton (1881), currently comprises two valid species: *P. cupulatus* (Eaton, 1871) from China (Ma et al. 2018; adults, larva, and egg) and *P. dao* Nguyen & Bae, 2004 from Vietnam (Nguyen and Bae 2004; larva only). Based on the unique characters of the imaginal and larval stages, Ma et al. (2018) clearly confirmed the generic status of *Paegniodes*, following Webb and McCafferty (2008). In addition, the complete mitochondrial genome of *P. cupulatus* was provided by Zhou et al. (2014).

In the past decade, knowledge about the diversity of the Heptageniidae in Thailand has continued to increase, and more species have been described and revised (Boonsoong and Braasch 2013; Boonsoong and Sartori 2015; Sutthacharoenthad et al. 2019). However, some genera of Thai heptageniid mayflies remain unclear and require taxonomic revision.

Here, we describe a new species of *Paegniodes* based on specimens from Nan province. In addition, the mitochondrial COI sequence data and a distribution map of the genus are provided.

## Materials and methods

*Paegniodes* larvae were collected by a hand-picking method from slow-flowing water in Nan Province in northern Thailand. The specimens were ﬁxed and preserved in 95% ethanol for molecular and morphological studies. Mature larvae were reared using earthenware pots connected to an air supply until emergence of winged stages.

Measurements (given in mm) and photographs were taken using a NIKON SMZ800 stereoscopic microscope. For scanning electron microscopy (**SEM**), eggs were dried in a critical point drier (CPD7501) and coated with gold (Sputter Coater SC7620). The SEM photographs were obtained with a FEI Quanta 450 SEM. Final plates were prepared with Adobe Photoshop CC 2020.

The preserved specimens were dissected for DNA extraction. Total DNA was extracted using a genomic DNA purification kit (NucleoSpin, Macherey-Nagel, Germany), following the manufacturer’s protocol. The COI amplification was performed using LCO1490 and HCO2198 (Folmer et al. 1994). The polymerase chain reaction (**PCR**) conditions and procedure were as described by Sutthacharoenthad et al. (2019). Purification and sequencing were conducted by Macrogen, Inc. (South Korea). The genetic distances between species were determined using Kimura-2-parameter distances (Kimura 1980), calculated with the MEGA X program (Kumar et al. 2018). Nucleotide sequences obtained in this study have been deposited in the GenBank database. Other *Paegniodes* sequences were also obtained from the Barcode of Life Data System (**BOLD**) and GenBank; details are presented in Table 1. The distribution map was generated with the SimpleMappr software (Shorthouse 2010).

**Table 1.** Sequenced specimens of the genus *Paegniodes* (new sequence indicated in bold).

Species	Locality	GenBank/BOLD Accession Number (GenSeq Nomenclature)
***P. sapanensis* sp. nov.**	**Nan, Thailand**	**MW633481 (genseq-2 COI)**
***P. sapanensis* sp. nov.**	**Nan, Thailand**	**MW633482 (genseq-2 COI)**
*P. dao* (BOLD identification)	Nan, Thailand	THMAY162
*P. cupulatus*	China	GBMH11533

The material is deposited in the collection of the Zoological Museum at Kasetsart University in Bangkok, Thailand (**ZMKU**) and at the Museum of Zoology in Lausanne, Switzerland (**MZL**).

## Taxonomy

### Order Ephemeroptera

**Family Heptageniidae**

### Genus *Paegniodes* Eaton, 1881

#### Paegniodes sapanensis

Taxon classificationAnimaliaEphemeropteraHeptageniidae

Boonsoong, Sartori & Auychinda sp. nov.

D9DDD476-96C7-5170-9A01-473ABD0C2E00

http://zoobank.org/1C749EC4-356B-4CCD-A22F-9C3AADD603AA

Figures 1 , 2 , 3 , 4 , 5 , 6 , 7 , 8 , 9 , 10

##### Materials examined.

***Holotype*.** 1 female mature larva in alcohol, deposited in ZMKU, Thailand, Nan province, Bo Kluea district, Sapan waterfall, 19°11'25.8"N, 101°11'56.3"E, 800 m, 21.III.2020, B. Boonsoong leg.

***Paratypes*.** 3 larvae in ethanol, deposited in ZMKU, same data as holotype; 2 larvae in ethanol, GBIFCH00834844, deposited in MZL same locality as holotype, 26.XI.2019, B. Boonsoong leg.; 1 male subimago (reared from larva), 2 female subimagos (reared from larvae), 2 larvae, all in ethanol, deposited in ZMKU, same locality as holotype, 29.XI.2020, B. Boonsoong leg.

##### Description of larva.

Body length 16.2 mm (holotype) 10.0–13.5 mm (exuvia) 7.2–11.5 mm (immature), caudal filaments ca 1.5× of body length (immature).

General colouration dark brown with pale markings on tibiae and abdominal tergites.

**Figure 1.**
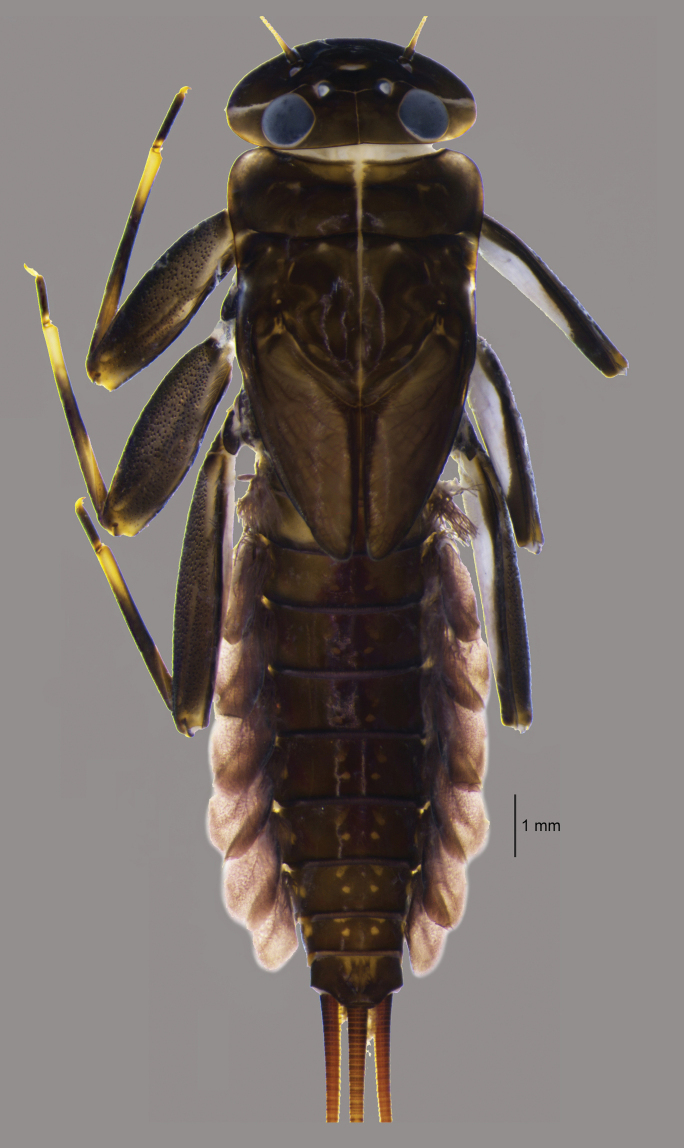
*Paegniodes sapanensis* sp. nov., habitus of larva (Holotype).

***Colouration*** (Figs 1, 10A–C). Head, thorax, legs, and abdomen dorsally dark brown. Head, thorax, legs, and abdomen ventrally whitish. Caudal filaments brown.

**Figure 2.**
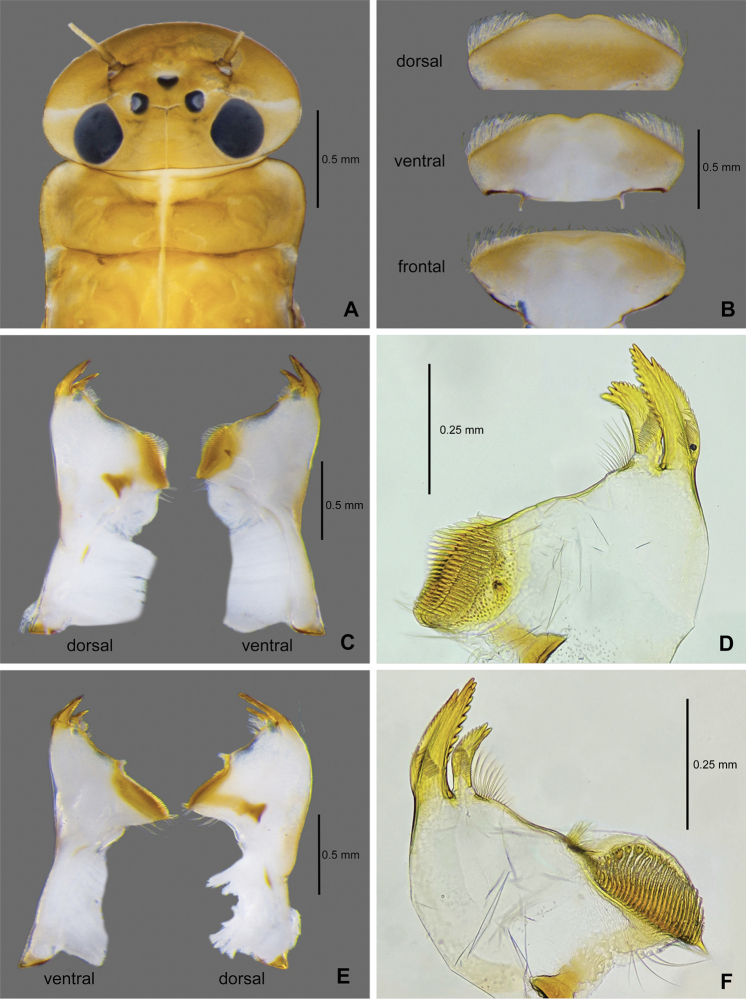
*Paegniodes sapanensis* sp. nov., larval morphology **A** head and pronotum **B** labrum **C** left mandible **D** enlargement of left mandible (ventral view) **E** right mandible **F** enlargement of right mandible (ventral view).

**Head. *Head capsule*.** Ovoid in shape and flattened, 1.8–2.5 mm in length, 2.5–4.1 mm in width, brown, without distinct markings (Fig. 2A); head capsule margins smooth. Compound eyes and base of ocelli black.

***Antenna*** (Figs 2A, 10A). Antennae length slightly longer than head width, scape and pedicel dark brown, flagellum light brown.

***Labrum*** (Fig. 2B). Triangular, width of labrum 1/3 of head capsule, with median notch on anterior margin, anterior margin with row of long, hair-like setae; dorsal surface with hair-like setae except at notch and nearby area.

***Left mandible*** (Fig. 2C). Outer and inner incisors acute with serrated margins, row of bristles located near inner incisor, row of fine serrated spines locate on base of outer and inner incisors, margin between inner incisor and mola slightly concave and smooth, one distinct denticle ventrally near mola area, setae present at apex of mola (Fig. 2D). Lateral margin without row of setae.

***Right mandible*** (Fig. 2E). Outer and inner incisors acute with serrated margins, row of bristles located near inner incisor, row of fine serrated spines locate on base of outer and inner incisors, margin between inner incisor and mola slightly concave, tuft of setae locate on inner margin near mola, setae present at apex of mola (Fig. 2F). Lateral margin without row of setae.

**Figure 3.**
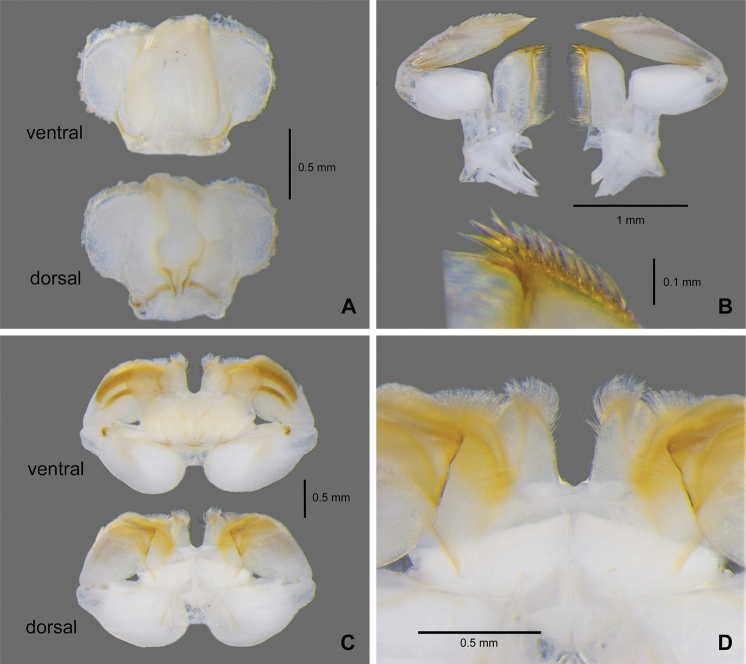
*Paegniodes sapanensis* sp. nov., larval morphology **A** hypopharynx **B** maxilla **C** labium (ventral view and dorsal view) **D** enlargement of labium (dorsal view).

***Hypopharynx*** (Fig. 3A). Lingua subequal to superlingua, longer than broad, with medial tuft of long, stout setae. Superlingua distally almost straight, nearly square; each superlingua with notch on anterior margin, lateral margin rounded, with fine, long, simple setae along laterodistal margin.

***Maxilla*** (Fig. 3B). Crown of galea-lacinia with a row of eight comb-shaped setae, apex of maxilla with three canines; two dentisetae, and one row of long setae on inner margin, with a row of submarginal setae on ventral surface; maxillary palpi two-segmented, apical one 1.6× length of basal segment; ventral surface of segment II with row of dense setae forming brush-like structure, apex of last segment apically lanceolate.

***Labium*** (Fig. 3C). Labium with U-shaped separation between glossae; shape of glossae conical, inner margin covered with dense setae, outer margin with row of setae; paraglossae moderately expanded laterally, with dense apical setae; basal segment of palp slightly longer than length of apical segment; apical segment slightly pentagonal, apex with broad projection (Fig. 3D); with dorsal transverse row of setae apically and setae brush ventrally.

**Figure 4.**
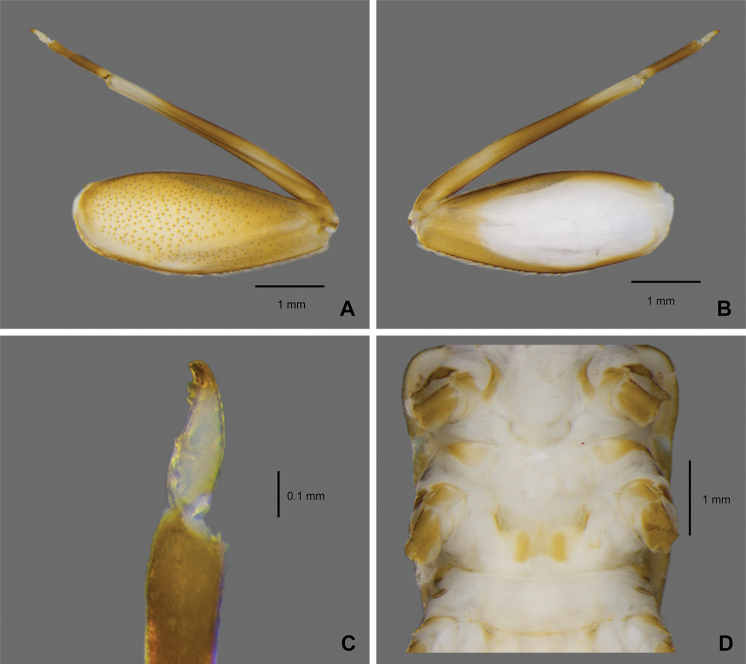
*Paegniodes sapanensis* sp. nov., larval morphology **A** foreleg (dorsal view) **B** foreleg (ventral view) **C** tarsal claw of foreleg **D** sternum.

**Thorax. *Foreleg*** (Fig. 4A) Coxa with well-developed, round dorsal plate; tibia subequal to femur in length, tarsus about ¼ length of tibia; femur with regular row of bristles on outer margin and many scattered, mostly spatulate setae on dorsal surface, ventrally with whitish elongated oval area (Fig. 4B); tibia and apex of tarsus pigmented in light yellow; tarsus brown to dark; claw with a submedian denticle and three apical denticles (Fig. 4C). ***Middle and hind legs*** as foreleg but with patellar-tibial (fusion) sutures, apex of tibia with cluster of setae on inner surface, and claw with two or three apical denticles. Mesosternum with a distinct transverse yellow macula (Fig. 4D).

**Abdomen. *Terga*** (Fig. 6A). Terga pale brown, each tergum with two submedian pairs of pale dots; mature larva with distinct brown median stripes on terga II–VII, with brown oblique stripes on lateral margin of terga III–VII, posterior margin of each tergum with row of strong and acute denticles, posterolateral projections extended into acute projections.

**Figure 5.**
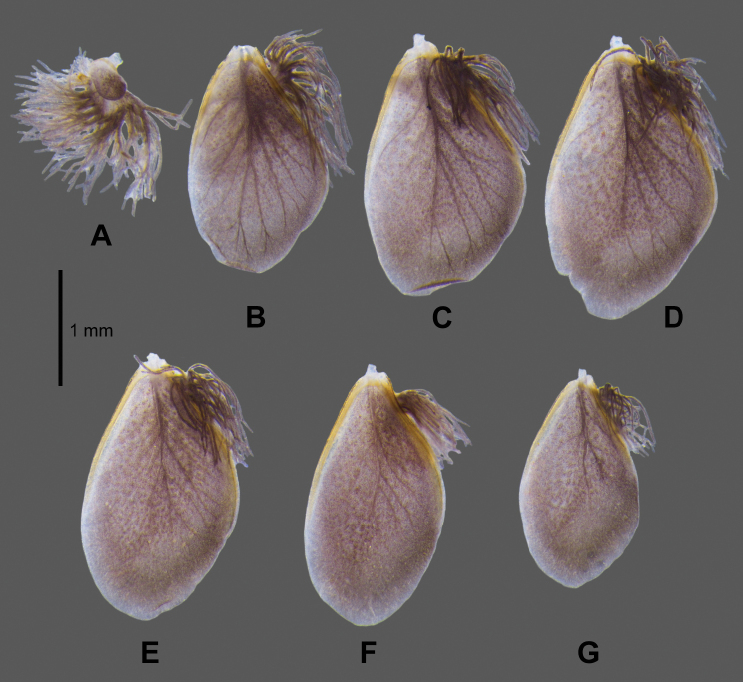
*Paegniodes sapanensis* sp. nov., larval morphology **A** gill I **B** gill II **C** gill III **D** gill IV **E** gill V **F** gill VI **G** gill VII.

***Gills*** (Fig. 5). Gills on abdominal segments I–VII; gill I (Fig. 5A) smaller than others, dorsal lamellae 1/4 in length of well-developed fibrilliform portion; gill II–VII similar in shape (Fig. 5B–G), lamellae much longer than fibrilliform portion, tracheation clearly visible, proximal half of lamellae margin thickened and sclerotised, gill IV (Fig. 5D) relatively larger than others.

**Figure 6.**
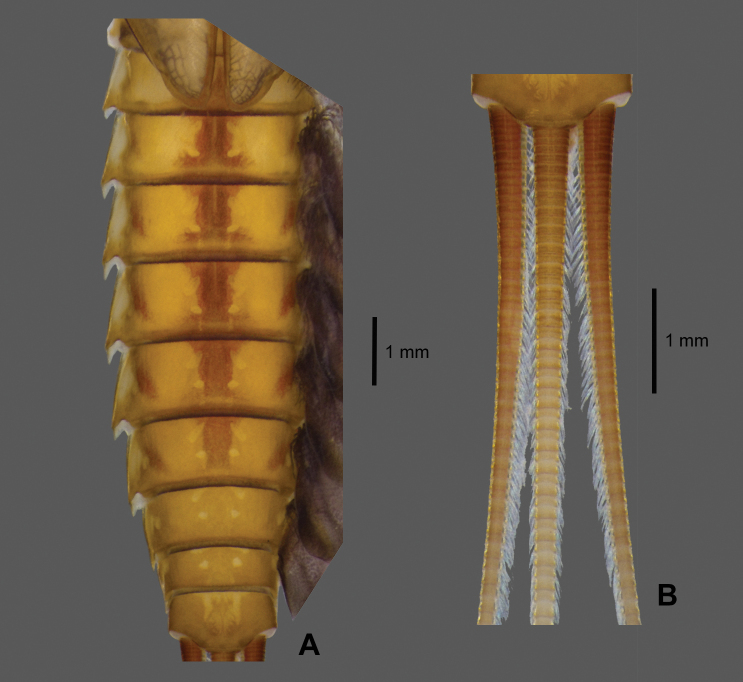
*Paegniodes sapanensis* sp. nov., larval morphology **A** terga II–X (dorsal view) **B** cerci and paracercus.

***Caudal filaments*** (Fig. 6B). Cerci subequal to paracercus in length, paracercus laterally with long setae on both margins of each segment, similar setae located on inner margins of cerci only.

##### Diagnostic characters of larval stage.

The main diagnostic characters are: i) lamellae of gill I ca 1/4 of fibrilliform portion, ii) mandibles without dense hair-like setae on lateral margin, iii) basal segment of maxillary palp without hairlike setae on margins, and iv) apical segment of labial palp slightly pentagonal with broad projection at apex.

##### Description of adult stages.

**Figure 7.**
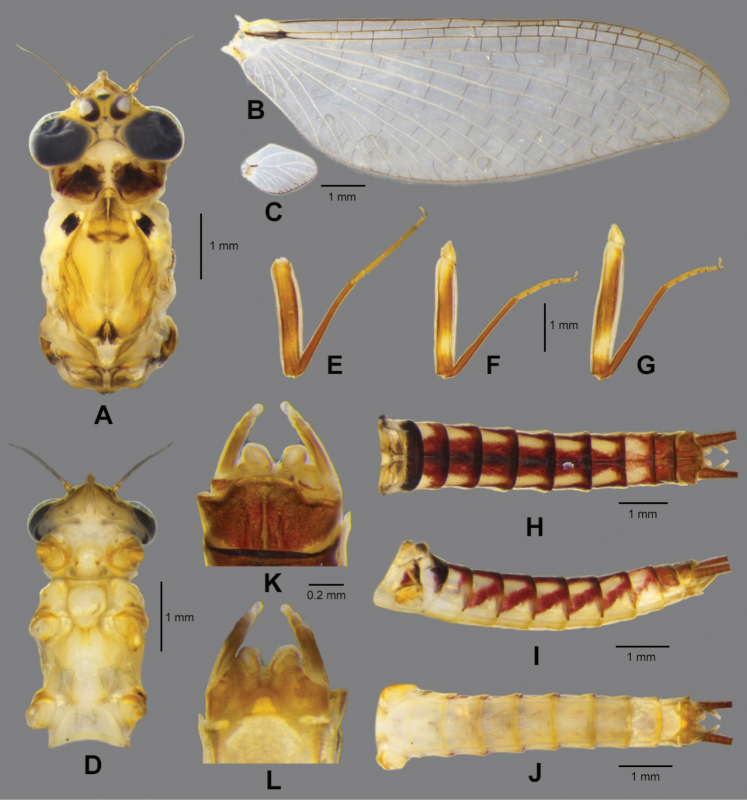
*Paegniodes sapanensis* sp. nov., male subimago **A** head and thorax (dorsal view) **B** forewing **C** hindwing **D** head and thorax (ventral view) **E** foreleg **F** middle leg **G** hind leg **H** abdomen (dorsal view) **I** abdomen (lateral view) **J** abdomen (ventral view) **K** genitalia (dorsal view) **L** genitalia (ventral view).

**Male subimago** (in ethanol Fig. 7, living Fig. 10E)

Body length 8.5 mm, cerci 17.5 mm, forewing 10.9 mm, hindwing 1.5 mm.

***Colouration*** (Fig. 7). Head, thorax and abdomen dorsally yellowish brown. Head, thorax, and abdomen ventrally light yellow. Legs yellow. Caudal filaments brownish.

***Head*** (Fig. 7A). Compound eyes separated by 3.0× width of median ocellus. ***Thorax*** (Fig. 7A, D). Pronotum and mesonotum each with pair of dark dots; forewings semitransparent, veins yellowish to brown (Fig. 7B); hindwings 0.14 size of forewings (Fig. 7C). Legs yellowish to yellowish brown; femora brown with proximal and distal light maculae; tibiae uniformly brown; tibiae slightly shorter than femora. Forelegs (Fig. 7E): length of leg segments: femur 2.5 mm; tibia 2.3 mm; tarsus 2.0 mm (tarsal segments in order of decreasing length: 2>3>4>1>5). Midlegs: (Fig. 7F) length of leg segments: femur 2.4 mm; tibia 2.1 mm; tarsus 1.2 mm (tarsal segments in order of decreasing length 2>1>5>3>4). Hindlegs (Fig. 7G): length of leg segments: femur 2.8 mm; tibia 2.2 mm; tarsus 1.2 mm (tarsal segments in order of decreasing length: 1≥5>2≥3>4). Each leg with two claws; one blunt, and one sharp and hooked.

***Abdomen*.** Dorsally with ornamentation as in Fig. 7H, tergum I with transverse dark band, terga II–VII with distinct, reddish median band; laterally with pattern as in Fig. 7I, with clearly oblique stripes on terga III–VII but those on terga II and VIII less visible; all sterna predominantly light yellow (Fig. 7J). Combined length of two terminal segments of gonopods half the length of basal one (Fig. 7K, L). Penis lobes jointed at base, apices separated, each penis lobe apex slightly expanded laterally. Styliger plate with concave posterior margin, but median part convex (Fig. 7L). Cerci reddish to brown.

**Figure 8.**
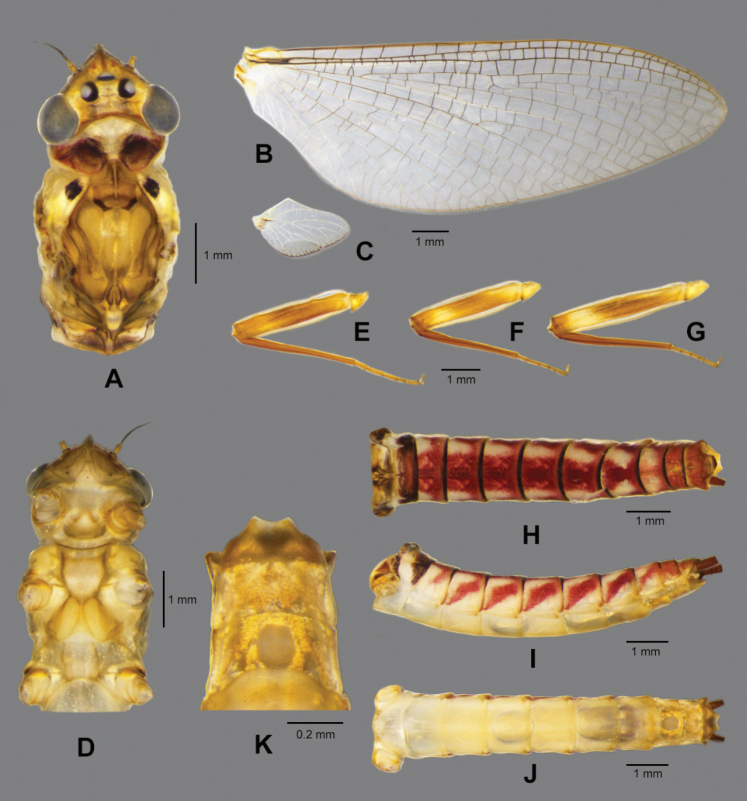
*Paegniodes sapanensis* sp. nov., female subimago **A** head and thorax (dorsal view) **B** forewing **C** hindwing **D** head and thorax (ventral view) **E** foreleg **F** middle leg **G** hind leg **H** abdomen (dorsal view) **I** abdomen (lateral view) **J** abdomen (ventral view) **K** genitalia (ventral view).

**Female subimago** (in ethanol Fig. 8, living Fig. 10D, F)

Body length 12.8 mm, cerci 20.0 mm, forewing 14.4 mm, hindwing 2.6 mm.

***Colouration*** (Fig. 8). Head, thorax and abdomen dorsally brown. Head, thorax and abdomen ventrally light brown. Legs yellow. Caudal filaments brown.

***Head*** (Fig. 8A). Compound eyes separated by 3.0× width of median ocellus.

***Thorax*** (Fig. 8A, D). Pronotum and mesonotum each with pair of dark dots, sternum light brown; forewings semitransparent, veins yellowish to brown (Fig. 8B); hindwings 0.18 the size of the forewings (Fig. 8C). Legs yellowish to yellowish brown; femora brown with proximal and distal light maculae; tibiae uniformly brown; foretibiae equal to femora; tibiae of midleg and hindleg slightly shorter than femora. Forelegs (Fig. 8E): length of leg segments: femur 3.1 mm; tibia 3.1 mm; tarsus 1.7 mm (tarsal segments in order of decreasing length: 2>3>5>1>4). Midlegs (Fig. 8F): length of leg segments: femur 2.9 mm; tibia 2.7 mm; tarsus 1.4 mm (tarsal segments in order of decreasing length 2>1≥3≥5>4). Hind leg (Fig. 8G): length of leg segments: femur 3.5 mm; tibia 3.0 mm; tarsus 1.2 mm (tarsal segments in order of decreasing length: 5>1≥2>3>4). Each leg with two claws; one blunt, and one sharp and hooked.

***Abdomen*.** Dorsally with ornamentation as in Fig. 8H, tergum I with dark band, with distinct reddish median band on terga II–VII; laterally with pattern as in Fig. 8I, with clearly oblique stripes on terga III–VII but those on terga II and VIII less visible; all sterna predominantly light yellow (Fig. 8J); subgenital plate (sternum VII) distally rounded and subanal plate (sternum IX) extended, with shallow median notch (Fig. 8K); cerci reddish brown, with tiny setae on surface.

##### Description of egg.

**Figure 9.**
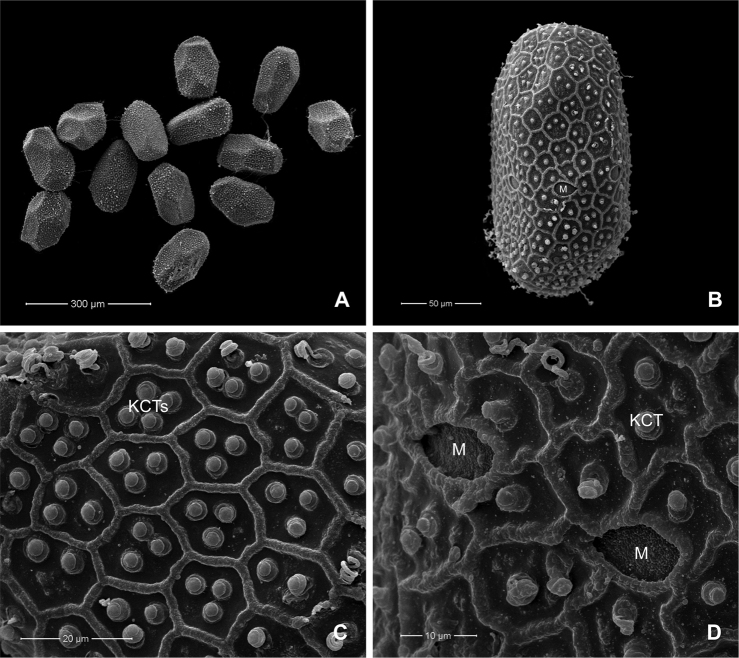
*Paegniodes sapanensis* sp. nov., scanning electron micrographs of egg (dissected from subimago) **A** eggs **B** general outline **C** chorionic surface **D** micropyle. M = micropyle, KCTs = knob-terminated coiled threads.

(dissected from female subimago). Length ca 155–175 µm, width ca 80–95 µm; elongate and oval in shape (Fig. 9A, B); chorionic surface covered with hexagonal and pentagonal mesh ridges, with 1–4 knob-terminated coiled threads (KCTs) in between (Fig. 9C); tagenoform micropyle in equatorial area (2 or 3 micropyles clearly visible on the same side) (Fig. 9D).

##### Diagnostic characters of imaginal stage.

The diagnostic characters to distinguish our new species from *P. cupulatus* are: i) the median stripes on abdominal terga and ii) lateral margins of genital plates slightly concave near apex.

**Figure 10.**
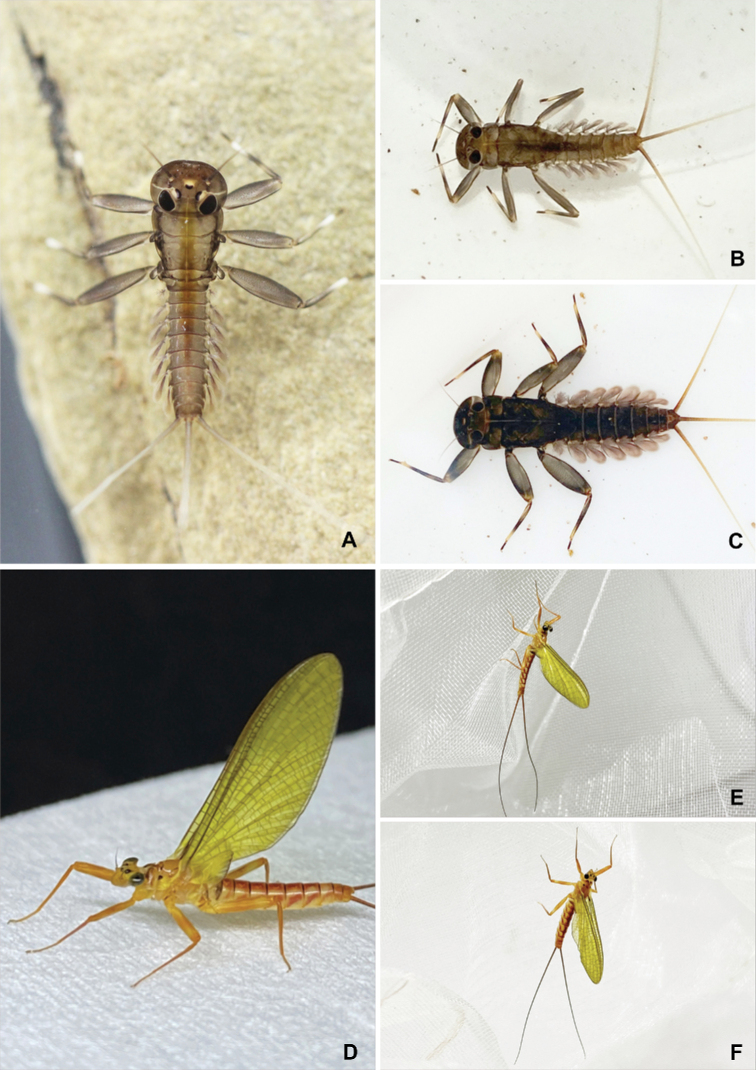
*Paegniodes sapanensis* sp. nov., habitus (live) **A** immature larva **B** male larva **C** female larva **D** closer view of female subimago **E** male subimago **F** female subimago.

##### Etymology.

The specific epithet is named for the Sapan waterfall (Bo Kluea district; tourist attraction of Nan province, Thailand), where the holotype is known.

##### Distribution.

Nan province.

##### Biological aspects.

**Figure 11.**
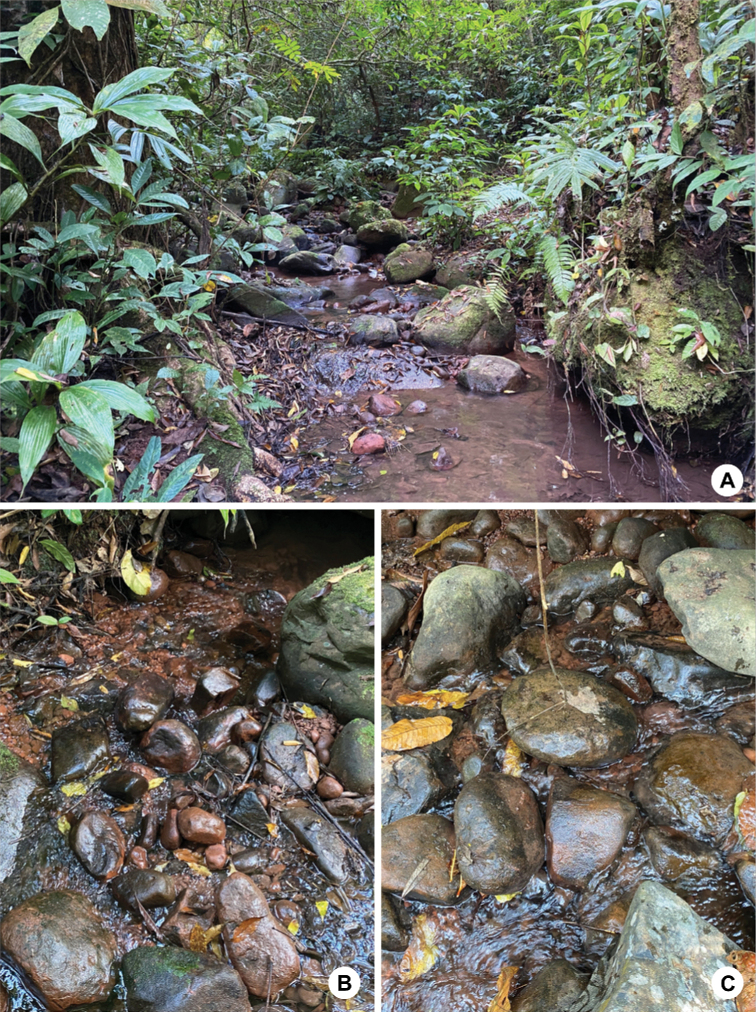
Habitats of larvae of *Paegniodes sapanensis* sp. nov. **A** tributary of a Sapan stream **B** cobble substrate with bottom sand and gravel **C** stream bank with cobble.

The specimens were collected from tropical mountain streams (Fig. 11A) which are slightly disturbed by tourist activities. The larvae of the new species were found in flowing areas and the littoral zone of the streams, underneath a mostly cobble substrate (Fig. 11B, C).

##### Molecular analysis.

Two COI sequences of *Paegniodes* were retrieved from BOLD system and GenBank (Table 1). One sequence from BOLD (THMAY162-12) was based on a specimen from the same locality (Namtok Sa Pan) as this study and this specimen was identified as *P. dao*. However, our Kimura 2-parameter (K2P) analysis revealed that intraspecific genetic divergence of the three sequences is very low (0.03%), and we considered all sequences as belonging to the same species. In addition, the interspecific distances (COI) between the new species and *Paegniodes cupulatus* ranged from 11.43–11.73%.

**Figure 12.**
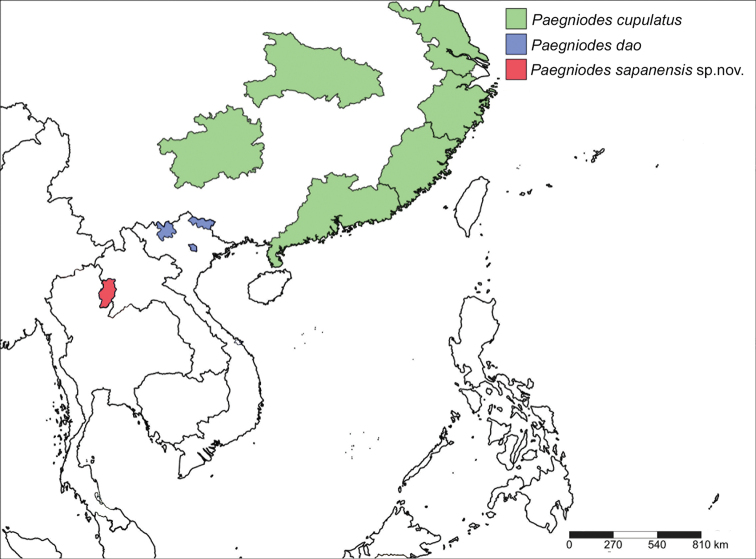
Distribution of the genus *Paegniodes* Eaton, 1881.

## Discussion

In this study, the morphology of the new species showed a close similarity to *P. cupulatus* (from China) in terms of mandibles, maxillary palp, gill I, and abdominal pattern (Table 2). Mandibles and basal segment of maxillary palp of *P. dao* (Nguyen and Bae 2004: fig. 1C, D) possess a dense, hair-like setal field on the lateral margin, which is not the case in *P. cupulatus* (Ma et al. 2018: fig. 4D, G) and *P. sapanensis* sp. nov. The dorsal lamellae of gill I of *P. cupulatus* and *P. sapanensis* sp. nov. represent at least 1/4 of a fibrilliform portion, while this is barely visible in *P. dao* (Nguyen and Bae 2004: fig. 1G). In addition, the larvae of *P. cupulatus* and *P. sapanensis* sp. nov. possess the typical abdominal stripes, which are absent in *P. dao* (Nguyen and Bae 2004).

**Table 2.** Larval characters of *Paegniodes sapanensis* sp. nov. compared with known species (Nguyen and Bae 2004; Ma et al. 2018).

Species	*P. cupulatus*	*P. dao*	*P. sapanensis* sp. nov.
Distribution	China	Vietnam	Thailand
Mandibles	without dense hairlike setal field on lateral margin	with dense hairlike setal field on lateral margin	without dense hairlike setal field on lateral margin
Basal segment of maxillary palp	without dense hairlike setal fields on anterior and posterior margins	with dense hairlike setal fields on anterior and posterior margins	without dense hairlike setal fields on anterior and posterior margins
Number of comb-shape setae on the crown of the galea-lacinia	9	unknown	8
Shape of labial palp segment II	apex with broad projection	apically rounded	apex with broad projection
Number of apical denticles of tarsal claw	3	unknown	2–3
Lamellae of gill I	1/3	rudimentary	1/4

However, the new species can be separated from *Paegniodes cupulatus* by the colouration of mature nymphs of *P. sapanensis* sp. nov. which seem darker than that of *P. cupulatus*. On the abdominal terga of *P. cupulatus*, the pale dots are on both side of median stripe (Ma et al. 2018: fig. 1A), while pale dots of the new species are inside the median stripe (Fig. 6A). Size of lamellate in gill I of the new species is smaller than in *P. cupulatus* (Table 2). The imaginal stage of the new species also differs from *P. cupulatus* by the width of the median stripe on abdominal terga; in *P. cupulatus* this is narrower than in the new species. Lateral margins of subanal plate are slightly concave near apex in the new species but smooth in *P. cupulatus* (Ma et al. 2018: figs 6D, 8F).

The molecular analysis clearly supports *P. sapanensis* sp. nov. as a species separate from *P. cupulatus*, as the genetic distance between the two species is 11%, which is much higher than the value of 3.5% generally considered to represent the maximum intraspecific divergence (Hebert et al. 2003; Zhou et al. 2010). The intraspecific genetic distance of the new species was about 0.03%, which is not surprizing, as all specimens were sampled in the same locality.

The combination characteristics of *Paegniodes* that distinguish it from all other genera in the subfamily Rhithrogeninae include: i) reduced lamellae on the gills I, ii) caudal filaments with interfacing setae, iii) short hindwings (usually less than 1/4 the length of the forewings), and iv) males having widely separated penes with strongly median titillators (Nguyen and Bae 2004; Webb and McCafferty 2008; Ma et al. 2018). The findings of this study revealed that the common characters in adults of *Paegniodes* were an abdomen with median and a pair of oblique stripes in both male and female subimagoes, as also found in *P. cupulatus* and *P. dao* (Nguyen and Bae 2004; Ma et al. 2018). Additionally, the unique egg chorionic structure of *Paegniodes* includes hexagonal mesh ridges and 1–4 knobs between them (Ma et al. 2018). Unlike the relatively similar *Rhithrogeniella* (subfamily Ecdyonurinae), only one KCT is found between the ridges (Kang and Yang 1994; Sartori 2014).

The distribution of the genus *Paegniodes* is limited to Southeast Asia (Fig. 12). This is the first discovery of *Paegniodes* in Thailand after a decade of Ephemeroptera investigations. The genus *Paegniodes* is local and rare in Thailand, probably due to precise ecological requirements. In this study, we found the larvae in a unique microhabitat, located in slightly disturbed and mountain streams of Nan province.

Ma et al. (2018) expressed the view that the position of *Paegniodes* is apparently a plesiomorphic lineage of the subfamily Rhithrogeninae based on unique characteristics. The mouthparts and genitalia of the genus *Paegniodes* are closely related to *Rhithrogena*, but more plesiomorphic. However, the eggs of *Paegniodes*, unlike any genus in the subfamily Rhithrogeninae, are somewhat similar to the genus *Rhithrogeniella* (Ecdyonurinae). Our finding supports the taxonomic status of the genus *Paegniodes* suggested by Ma et al. (2018).

## Supplementary Material

XML Treatment for Paegniodes sapanensis
